# Vibration Analysis of Al–Al_2_O_3_ Micro-Cantilever Sandwich Beams with Porosity in Fluids

**DOI:** 10.3390/mi16020206

**Published:** 2025-02-11

**Authors:** Feixiang Tang, Xiong Yuan, Siyu He, Jize Jiang, Shaonan Shi, Yuhan Li, Wenjin Liu, Yang Zhou, Fang Dong, Sheng Liu

**Affiliations:** 1 Key Laboratory of Transients in Hydraulic Machinery, Ministry of Education, School of Power and Mechanical Engineering, Wuhan University, Wuhan 430072, China; thomas0209@whu.edu.cn (F.T.); 2021302191690@whu.edu.cn (J.J.); 2021302191052@whu.edu.cn (S.S.); dragon0105@whu.edu.cn (Y.L.); lwj2004@whu.edu.cn (W.L.); 2 Hefei Archimedes Electronic Technology Co., Ltd., Hefei 230088, China; yang.zhou@ac-semi.com; 3 China-EU Institute for Clean and Renewable Energy, Huazhong University of Science & Technology, Wuhan 430074, China; 4 The Institute of Technological Sciences, Wuhan University, Wuhan 430072, China

**Keywords:** vibration analysis, pores distribution, scale effects, micro-cantilever beam, FGM sandwich

## Abstract

The vibration of porous Al–Al_2_O_3_ micro-cantilever sandwich beams in fluids was studied utilizing the modified couple stress theory and the scale distribution theory (MCST and SDT). Four types of porosity distributions were defined; the uniform distribution of pores was defined as U-type, while O-type, V-type and X-type represented non-uniform distributions of pores. The material properties of different porous sandwich beams were calculated. The properties of the micro-cantilever sandwich beams were adjusted to account for scale effects according to MCST. With the fluid driving force taken into consideration, the amplitude-frequency response, and resonant frequencies of the FGM sandwich beams in three different fluids were calculated using the Euler–Bernoulli beam theory. The computational studies showed that the presence of gradient factor p and the pores in the micro-cantilever sandwich beams affect the temperature field distribution and amplitude-frequency response in fluids. Increasing gradient factor p leads to a more obvious thermal concentration of the one-dimensional temperature field and migrates the resonance peaks to lower frequencies. In contrast to the uniform distribution type, the non-uniformly distributed pores also cause a decrease in the resonance frequency.

## 1. Introduction

Micro-electro-mechanical systems (MEMS) have seen significant advancements [1,2,3,4,5] in many fields in recent years, which has exacerbated the demand for higher-performance materials. There is a consensus that composite structures are playing an increasingly significant role in modern science and technology. Traditional composite structures are usually built in a multi-layer form with apparent interfaces between layers, which causes discontinuities at the interfaces. The delamination between material phases often causes the failure of conventional composite structures and is a significant drawback.

Functionally graded material (FGM) structures, which gradientally vary in material properties from one surface to another, offer unique advantages in terms of strength, resonance properties, and environmental resistance, overcoming the drawback of conventional composite structures [6].

However, many experiments have shown that when the dimensions are at the micro-nano level [7,8,9], the mechanical behavior of the structures shows distinct differences from those at the macro level. Many scientists have conducted research on this size-dependent effect. Fleck found that the slopes of the stress–strain curves of copper increase rapidly with decreasing diameters. Lam et al. (2003) [10] discovered that micro-beams with a thickness of 20 μm exhibited an equivalent stiffness that was over two times greater than those with a thickness of 115 μm. Lei et al. (2016) [11] observed that subtracting the thickness of beams from 15 μm to 2.1 μm consistently resulted in a measured vibration frequency that surpassed the predictions of the classical model of beams. In 2018, Li et al. (2018) [12] conducted a study on the dynamic behavior of micro-scale beams.

The conventional continuum theory is not capable of characterizing the scale effect. To better describe the scale effect, scientists have proposed various theories such as the strain gradient theory and the non-local theory. Yang et al. [13] first presented a modified couple-stress theory that contains one single parameter about the size effect. The modified couple-stress theory considers both the displacement and rotation that occur together during the deformation process. This theory integrates the concept of material point rotation, which is derived from the traditional theory of continuous media. In 2014, Jung et al. [14] conducted research on the bending and vibration behaviors of FGM micro-plates supported by a Pasternak elastic foundation, employing the MCST. In the same year, Jani and Reddy [15] investigated the physical characteristics of web–core sandwich plates using the MCST. Farokhi and Ghayesh [16], in 2019, dedicated their work to developing a comprehensive formulation of the MCST for general orthogonal curvilinear coordinate systems. In 2020, Zahra et al. [17] explored the physical properties of multi-layer graphene sheets. Vinh [18] conducted a study of the vibration, buckling, and bending behaviors of two-dimensional FGM sandwich plates by employing the finite element method (FEM) based on the higher-order shear deformation theory (HSDT). Jiang et al. [19,20] studied the vibrations of Cu–Si beams with pores in fluids via MCST and investigated the vibration of functionally graded beams with physical neutral plane considerations. Shi et al. [21] analyzed the vibration of $Al–Al_{2}O_{3}$ microplates, incorporating neutral plane and scale effects. Wang et al. [22] studied the vibration and stability of an FG axis in an annular fluid.

In this study, we raise four distinct pore configurations in cantilever sandwich beams, building on research into the forced vibrations of micro-cantilever sandwich beams in various fluids under the impact of size effects. The four pore structures can be categorized into two types: uniformly distributed (U-type) and non-uniformly distributed (O-type, V-type, and X-type). We examine the material properties of $Al–Al_{2}O_{3}$ micro-cantilever sandwich beams with a power-law distribution approach. We formulate the one-dimensional thermal distribution within a micro-scale cantilever sandwich beam subjected to laser stimulation and computationally determine the vibration responses of these beams to thermal and hydrodynamic stimuli using the Euler–Bernoulli theory and Galerkin’s method. A numerical analysis also discussed how pore distribution type, scale effects, gradient factors, fluid characteristics, and geometry impact the Young’s modulus and resonant frequency of the sandwich beams, which is pertinent to mass sensing and fluid characterization applications.

## 2. Theoretical Analysis

### 2.1. Power-Law Model of Porous Sandwich Micro-Cantilever Beam

**Figure 1:**
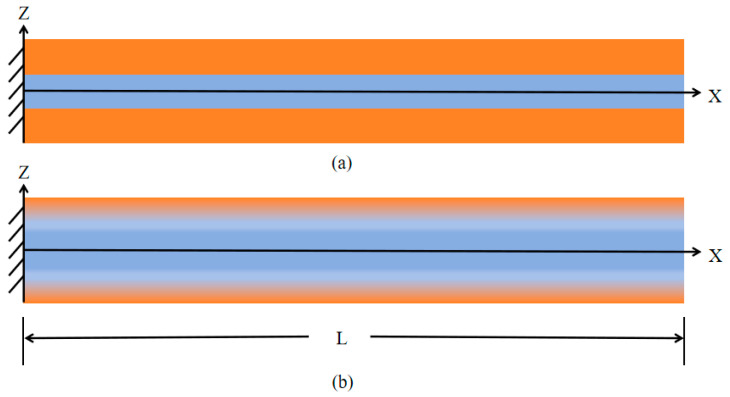
The schematics of: (**a**) composite micro-cantilever beam; and (**b**) FGM sandwich micro-cantilever beam.

FGM cantilever sandwich beams are composed of two materials. Unlike conventional composite structures, there are no distinct interfaces in FGM sandwich structures, as shown in Figure 1. In this research, the top of the beam is defined as Al and the core is defined as $Al_{2}O_{3}$. The detailed physical properties of the relevant materials are listed in Table 1.

**Figure 2:**
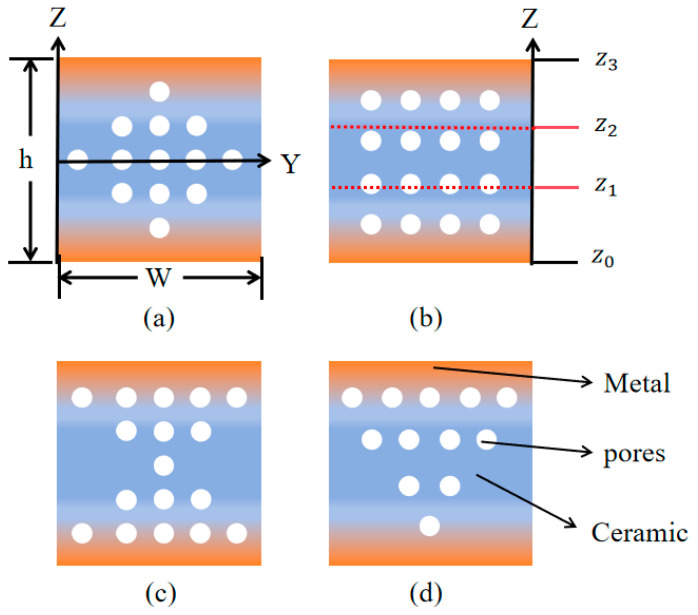
Cross-sections of FGM sandwich micro-cantilever beams with: (**a**) O-type distributed porosity; (**b**) U-type distributed porosity; (**c**) X-type distributed porosity; and (**d**) V-type distributed porosity.

In Figure 2, we define four kinds of porous FGM sandwich beams with, respectively, O-type, U-type, X-type, and V-type distributed porosity. The sandwich beams have an upper layer ([z_2_–z_3_]) and a bottom layer ([z_0_–z_1_]) of FG material and a core layer ([z_1_–z_2_]) of pure ceramic material. For the upper and bottom FGM layer, the material is functionally graded material made of metal (marked in orange) and ceramic (marked in blue). The dimensions of the beams are defined as: the height h, the width W, and the length L. The four pore structures can be categorized into two types: uniformly distributed (U-type) and non-uniformly distributed (O-type, V-type, and X-type). The porosity volume of the plate is Vp*. The porosity varies across the Z-direction and are isotropic in the X-direction and Y-direction.

**Figure 3:**
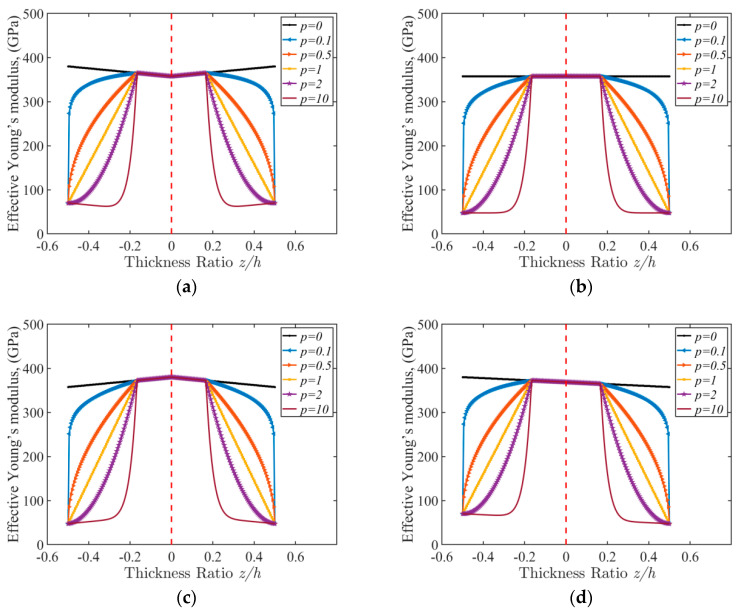
Effective Young’s Modulus of FGM sandwich cantilever beams with: (**a**) O-type distributed porosity; (**b**) U-type distributed porosity; (**c**) X-type distributed porosity; (**d**) V-type distributed porosity, $V_{p}$ = 0.1. The red dash stands for the plane with z-coordinate equalling to zero.

In Figure 3, the effective Young’s modulus ($E^{*}=\int_{z_{0}}^{z_{3}}E(z)dz$) of the FGM sandwich beams with different power-law parameters *p* are shown. The volume of the porosity is defined as 0.1. For all four types of FGM sandwich beams, the parameter p has a significant influence on the distribution of the Young’s modulus. When p equals to 0, the FGM material degrades into a pure material, as follows:

(1) $$\begin{array}{l} V_{c}=\left\{\begin{array}{cc} {\left(\frac{z-z_{0}}{z_{1}-z_{0}}\right)}^{p}, & z\in [z_{0},z_{1}]\\ 1, & z\in [z_{1},z_{2}]\\ {\left(\frac{z-z_{3}}{z_{2}-z_{3}}\right)}^{p}, & z\in [z_{2},z_{3}] \end{array}\right.\\ V_{m}=1-V_{c}. \end{array}$$

(2) $$P(z)=V_{c}P_{c}+V_{m}P_{m}-V_{p}\frac{\left(P_{c}+P_{m}\right)}{2}$$

For different types of pores, the *Vp* has the following diverse definitions:

(3) $$\begin{array}{l} V_{Op}=V_{p}^{*}\left|\frac{2z}{H}\right|;\\ V_{Xp}=V_{p}^{*}\left(1-\left|\frac{2z}{H}\right|\right);\\ V_{Up}=V_{p}^{*};\\ V_{Vp}=V_{p}^{*}\left(1+\frac{z}{H}\right); \end{array}$$

where $V_{Op}$, $V_{Op}$, $V_{Op}$ and $V_{Op}$, respectively, are the volume fraction of O-Type, X-Type, U-Type, and V-Type porosity in the FGM sandwich beams and Vp* is the porosity volume fraction.

According to Equation (2), we can obtain the material properties of four types of porous FGM sandwich beams such as the elasticity modulus, Poisson’s ratio, density, and so on. As demonstrated in Figure 3, the distribution of Young’s modulus through the z-direction of four types of porous FGM sandwich beams were calculated. The gradient factor p represents the percentage of the two materials (Al and $Al_{2}O_{3}$) in the beam and has a large impact on the distribution of material properties. Comparing the four distributions, we can see that where the pores are concentrated, they cause a decrease in the material properties.

### 2.2. One-Dimensional Temperature Field

**Figure 4:**
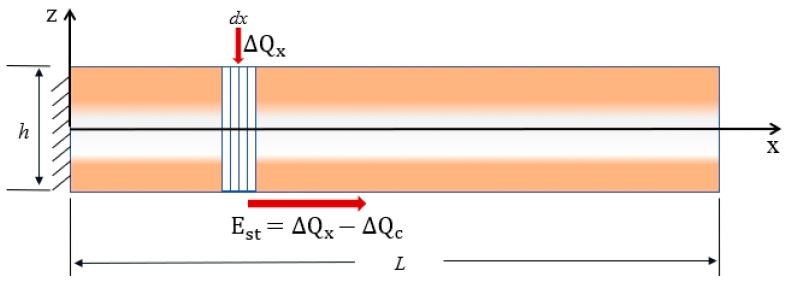
Temperature field model in the x-axis direction of the FG sandwich micro-cantilever beam.

In the temperature field model shown in Figure 4, we defined the microelement dx as the laser excitation for thermal conductivity studies. dx partially acquires heat upon excitation as $\Delta Q_{x}$ and has a temperature of T. The ambient temperature is defined as $T_{f}$. Due to the presence of a temperature difference, there is an exchange of heat between the cantilever beam and the fluid. The heat transferred is $\Delta Q_{c}$, assuming a heat transfer coefficient of γ. $E_{st}$ is the final energy storage term. W represents the width of the sandwich beam, and h represents the thickness. $\kappa_{eff}$, $\rho_{eff}$, and $C_{eff}$, respectively, represent the equivalent thermal conductivity, equivalent density, and equivalent specific heat capacity of the beam. According to the classical heat transfer theory the temperature field distribution equation can be derived as follows:

(4) $$\Delta Q_{x}=W\cdot \kappa_{eff}\cdot dx$$

(5) $$\Delta Q_{c}=2\gamma \cdot \left(W+h\right)\left(T-T_{f}\right)dx$$

(6) $$E_{st}=\rho_{eff}C_{eff}Wh\cdot \frac{\partial T}{\partial t}dx$$

(7) $$E_{st}=\Delta Q_{x}-\Delta Q_{c}$$

By substituting Equations (4)–(6) into Equation (7), we derive the one-dimensional temperature field distribution equation for the FG sandwich micro-cantilever beam, as follows:

(8) $$\frac{\partial \Delta T}{\partial t}=K\frac{\partial^{2}\Delta T}{\partial x^{2}}-\beta \Delta T$$

where ΔT, K and R can be expressed as follows:

(9) $$\Delta T=T-T_{f}$$

(10) $$K=\frac{\kappa_{eff}}{\rho_{eff}C_{eff}}$$

(11) $$\beta =\frac{2\gamma \cdot \left(W+h\right)}{W\rho_{eff}C_{eff}}$$

The Fourier transform of Equation (8) is as follows:

(12) $$\Delta \hat{T}\left(x,\omega \right)=\int_{-\infty }^{+\infty }\Delta T\left(x,t\right)e^{-i\omega t}dt$$

The time-domain control equations for the temperature field are transformed into frequency-domain control equations, as follows:

(13) $$\frac{\partial^{2}\Delta \hat{T}\left(x,\omega \right)}{\partial x^{2}}-\frac{\beta +i\omega }{K}\Delta \hat{T}\left(x,\omega \right)=0$$

where $∆\hat{T}$ is the temperature increment in the frequency domain. The absorption of laser energy by the cantilever beam is λ, and P is the laser power. The boundary conditions of thermal fields are established as follows:

(14) $$∆\hat{T}\left(x_{0}^{-}\right)=∆\hat{T}\left(x_{0}^{+}\right)$$

(15) $${\left.\frac{\partial ∆\hat{T}}{\partial x}\right|}_{x=x_{0}^{+}}-{\left.\frac{\partial ∆\hat{T}}{\partial x}\right|}_{x=x_{0}^{-}}=-\frac{\lambda P_{0}}{W\kappa_{eff}}$$

(16) $${\left.∆\hat{T}\right|}_{x=0}=0,{\left.\frac{\partial ∆\hat{T}}{\partial x}\right|}_{x=L}=-H∆\hat{T}$$

(17) $$H=\frac{\gamma h}{\kappa_{eff}}$$

where the temperature on the cantilever beam at the laser excitation should satisfy the continuity condition, as in Equation (14). Equation (15) means that the thermo-fluid temperature satisfies the quantum leap condition at $x_{0}$. The heat transfer boundary conditions was formulated in Equation (16). It is presumed there is no heat flow loss at the fixed end and the free end exhibits free heat dissipation. We can obtain the solution of Equation (8) after Fourier transform as follows:

(18) $$\left\{\begin{array}{c} ∆\hat{T}\left(x,\omega \right)=C_{1}e^{rx}+C_{2}e^{-rx},\left(x<x_{0}\right)\\ ∆\hat{T}\left(x,\omega \right)=C_{3}e^{rx}+C_{4}e^{-rx},\left(x\geq x_{0}\right) \end{array}\right.$$

where ω stands for circular frequency and r is the simplified parameter in the complex plane. These can be expressed as follows:

(19) ω=2πf

(20) $$r=\sqrt{\frac{\beta +\sqrt{\beta^{2}+\omega^{2}}}{2K}}+i\sqrt{\frac{-\beta +\sqrt{\beta^{2}+\omega^{2}}}{2K}}$$

The generalization coefficients $C_{1}\sim C_{4}$ in Equation (18) are determined by the boundary conditions of the Fourier transform. From Equations (14)–(16) the derivation can be derived as follows:

(21) $$\left\{\begin{array}{l} C_{1}=\frac{\lambda P_{0}}{W\kappa_{eff}}\times \frac{e^{-rx_{0}}\cdot \left[e^{2rL}\left(r+H\right)+e^{2rx_{0}}\left(r-H\right)\right]}{2r\left[e^{2rL}\left(r+H\right)+\left(r-H\right)\right]}\\ C_{2}=-C_{1}\\ C_{3}=\frac{\lambda P_{0}}{W\kappa_{eff}}\times \frac{e^{-rx_{0}}\cdot \left(e^{2rx_{0}}-1\right)\left(r-H\right)}{2r\left[e^{2rL}\left(r+H\right)+\left(r-H\right)\right]}\\ C_{4}=\frac{C_{3}\cdot e^{2rL}\left(r+H\right)}{\left(r-H\right)} \end{array}\right.$$

### 2.3. Analysis of Dynamic Response

A combination of the thermally driving force $F_{d}$ and the fluid driving force $F_{f}$ affects the FGM sandwich micro-cantilever beams when it is vibrated with laser excitation loaded in the fluids, as follows:

(22) $$F_{e}\left(x,\omega \right)=F_{d}\left(x,\omega \right)+F_{f}\left(x,\omega \right)$$

Because of the varying material properties of the FGM along the z-axis, the cantilever beam experiences asymptotic axial stresses along the thickness direction. Based on thermo-elasticity principles, the distribution of thermal stress and the bending moment along the x-axis for the FGM sandwich beam can be formulated as detailed below:

(23) $$\sigma \left(z\right)=E\left(z\right)\beta \left(z\right)\Delta \hat{T}\left(x,\omega \right)$$

(24) $$M\left(x,\omega \right)=W\int_{-\frac{h}{2}}^{\frac{h}{2}}\sigma \left(z\right)\left(z-z_{0}\right)dz$$

The axial bending moment has a dual-differential connection with the shear force according to classical theory. Then, $F_{d}$ can be obtained as follows:

(25) $$F_{d}=-\frac{\partial^{2}M\left(x,\omega \right)}{\partial x^{2}}=-W\int_{-\frac{d}{2}}^{\frac{d}{2}}E\left(z\right)\beta \left(z\right)\left(z-z_{0}\right)\times \frac{\partial^{2}\Delta \hat{T}\left(x,\omega \right)}{\partial x^{2}}dz$$

For the fluid driving force $F_{f}$ is obtained as follows [23]:

(26) $$F_{hydro}=\frac{\pi }{4}\rho_{f}W\omega^{2}\Gamma \left(\omega \right)Z\left(x,\omega \right)$$

where $\Gamma_{circ}\left(\omega \right)$ is the fluidics function of the beam with the circular section. $\Omega \left(\omega \right)$ is the corrective function for the cross-section. This can correct the value of the fluidics function from a circular section into a rectangular one.

After the equivalent force $F_{e}\left(x,\omega \right)$ of the beam is obtained, the deformation field $Z\left(x,\omega \right)$ can be obtained as follows:

(27) $${\left(EI\right)}_{eff}\frac{\partial^{4}Z\left(x,\omega \right)}{\partial x^{4}}-\rho_{eff}A\omega^{2}Z\left(x,\omega \right)=F_{e}\left(x,\omega \right)$$

where ${\left(EI\right)}_{eff}$ denotes the equivalent bending stiffness and A is the cross-sectional area.

(28) $$\left\{\begin{array}{c} A=W\times h\\ {\left(EI\right)}_{eff}=W\cdot \int_{-\frac{h}{2}}^{\frac{h}{2}}E\left(z\right){\left(z-z_{0}\right)}^{2}dz \end{array}\right.$$

Employing Galerkin’s method, the solution can be represented in the form of a free cantilever beam normalized to the amplification factor. Within the framework of Galerkin’s method, the trial function $\varphi_{i}\left(x\right)$ is defined by the boundary conditions specific to the cantilever beam. The frequency-dependent coefficient is the parameter that should be ascertained, as follows:

(29) $$\varphi_{i}\left(x\right)=a_{i}\left\{\cos\left(k_{i}x\right)-\cosh\left(k_{i}x\right)-\frac{\cos\left(k_{i}L\right)+\cosh\left(k_{i}L\right)}{\sin\left(k_{i}L\right)+\sinh\left(k_{i}L\right)}\left[\sin\left(k_{i}x\right)-\sinh\left(k_{i}x\right)\right]\right\}$$

(30) $$\left\{\begin{array}{c} a_{1}=\frac{1.000000054966522}{\sqrt{L}}\\ a_{2}=\frac{1.0000000424921067}{\sqrt{L}}\\ a_{3}=\frac{1.0000000837026268}{\sqrt{L}} \end{array}\right.$$

(31) $$\left\{\begin{array}{c} k_{1}=\frac{1.875104}{L}\\ k_{2}=\frac{4.694091}{L}\\ k_{3}=\frac{7.854757}{L} \end{array}\right.$$

where $k_{i}$ is the coefficient determined by the order function. In this study, we extend our calculations to the third order to determine the vibrational behavior of the cantilever beam. Thus, $a_{i}$ and $k_{i}$ are considered as the third order.

The dynamical deformation field $Z\left(x,\omega \right)$ can be solved as follows:

(32) $$Z\left(x,\omega \right)=\sum_{n=1}^{\infty }A_{n}\left(\omega \right)\varphi_{i}\left(x\right)$$

(33) $$A\left(\omega \right)=\frac{\int_{0}^{L}F_{drive}\left(x,\omega \right)\varphi_{i}\left(x\right)dx}{{\left(EI\right)}_{eff}\left\{\int_{0}^{L}{\left[\frac{d^{2}\varphi_{i}\left(x\right)}{dx^{2}}\right]}^{2}dx-\frac{\rho_{eff}A\omega^{2}}{{\left(EI\right)}_{eff}}\left[1+\frac{\pi W^{2}\rho_{f}}{4A\rho }\Gamma \left(\omega \right)\right]\right\}}$$

### 2.4. First Order Resonant Frequency

In Section 2.3, we presented the procedure for solving Euler’s Bernoulli equation to achieve a third-order, accurate, amplitude-frequency response for the FG sandwich micro-cantilever beam, employing Galerkin’s method [24]. Referring to the research, we obtained the resonant frequency of the micro-cantilever beam with a more computationally efficient formula, which is presented as follows:

(34) $$f_{0}=1.875^{2}\times \frac{1}{2\pi L^{2}}\sqrt{\frac{E_{eff}h^{2}}{12\rho_{eff}}}$$

## 3. Results Analysis

We examined the amplitude-frequency characteristics of FG cantilever sandwich beams when they are immersed in gasoline, air, and water, respectively.

The beams are made of FGM $Al–Al_{2}O_{3}$ and the material parameters of the beam and fluids are listed in Table 1 and Table 2.

In this section, we use a micro-cantilever beam with the following dimensions: L=300 μm; W=30 μm; and h=10 μm. The frequency of the loading laser is $f_{l}=17,430\;Hz$ and the power is $P_{0}=10\;W$. The loading location is $x_{0}=0.5\;L$. The coefficient of convective heat transfer from a gas to a solid surface is $\gamma =10\;W/(m^{2}\cdot \;K)$. The laser energy absorption coefficient of the micro-cantilever beams is defined as: λ=0.3. Micro-cantilever beams operate as a fluid circumstance of air. Under the condition of determining the porosity $V_{p}$ = 0.1, we investigated the thermal field of the beam with uniformly distributed pores at different gradient factors p.

**Figure 5:**
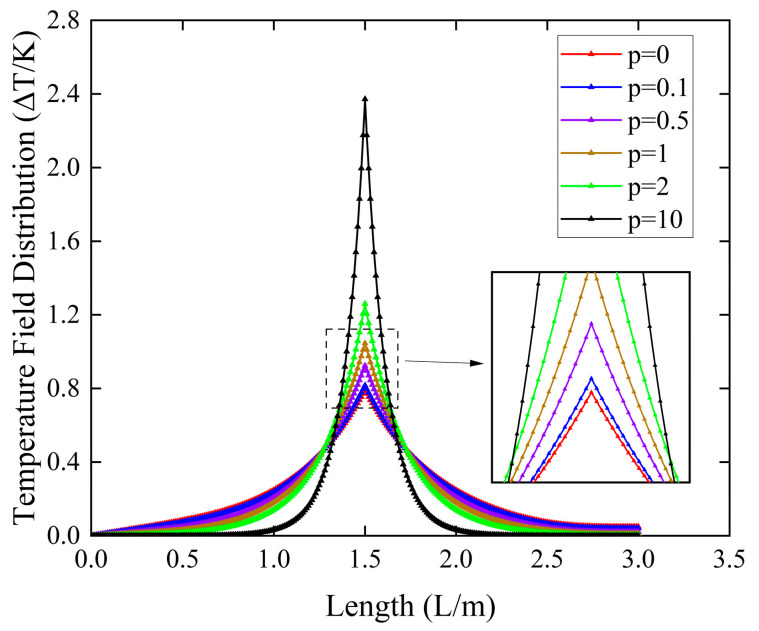
Thermal field distributions of micro-cantilever beams with diverse power-law parameters p.

In Figure 5, we can see that the temperature of the micro-cantilever beams is more centralized at $z_{0}$ as the gradient factor p increases. The diffusion of heat to the ends decreases because the increase in the gradient factor p increases the volume percentage of ${Al}_{2}O_{3}$ in the FGM sandwich micro-cantilever beams, leading to a rapid decrease in the thermal conductivity of the whole beam.

**Figure 6:**
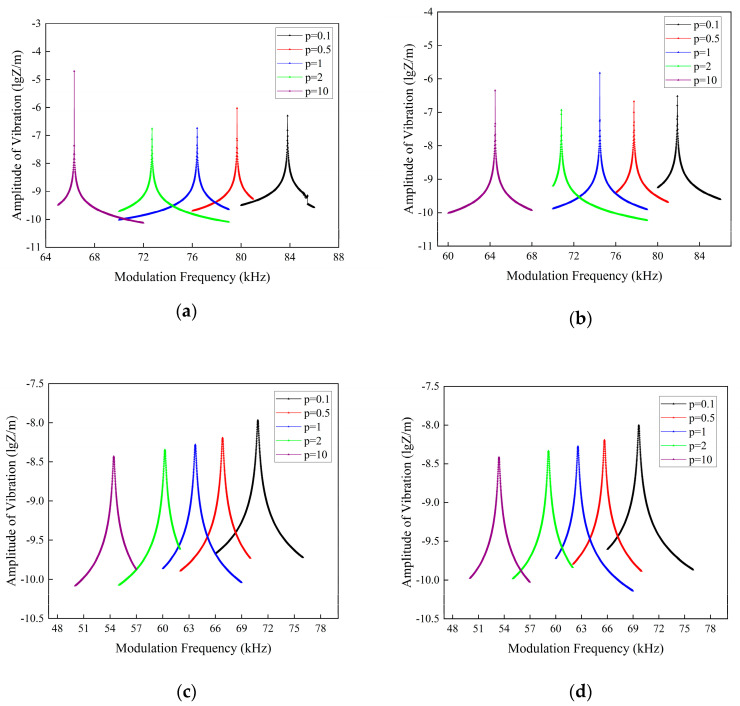

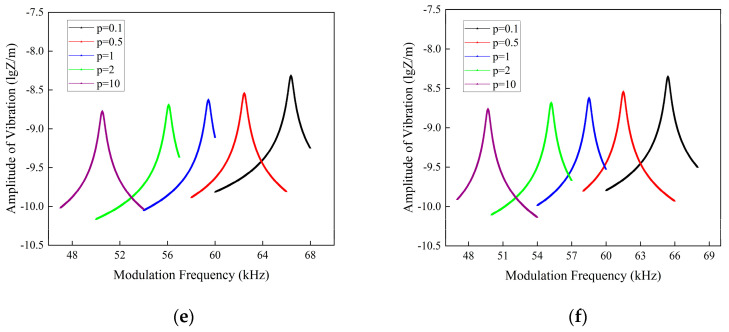
Amplitude-frequency response spectra of FG sandwich cantilever beams with various fluids and porosity: (**a**) even-type distributed porous beams in air; (**b**) uneven-type distributed porous beams in air; (**c**) even-type distributed porous beams in gasoline; (**d**) uneven-type distributed porous beams in gasoline; (**e**) even-type distributed porous beams in water; and (**f**) uneven-type distributed porous beams in water.

In Figure 6, we use micro-cantilever beams with the following dimensions: L=2500 μm; W=400 μm; and h=160 μm. The laser is loaded at $x_{0}=0.5\;L$ with a power of $P_{0}=10\times 10^{-3}\;W$. The amplitude-frequency response of the sandwich micro-cantilever beam in three diverse fluids was studied. In the longitudinal comparison, we can see that the presence of fluid causes the resonance peak of the beam to slow down and the resonance frequency to migrate to lower frequencies. The higher the kinematic viscosity of the fluid, the more pronounced this phenomenon is. This is due to the additional force $F_{f}$ of resistance created by the fluid as the cantilever beam vibrates. Making a side-by-side comparison for each curve, we can see that the resonant frequency of the sandwich FGM beam shrinks as the gradient factor p increases. This is due to the increase in the ${Al}_{2}O_{3}$ component of the sandwich micro-cantilever beam, which diminishes the equivalent Young’s modulus.

**Figure 7:**
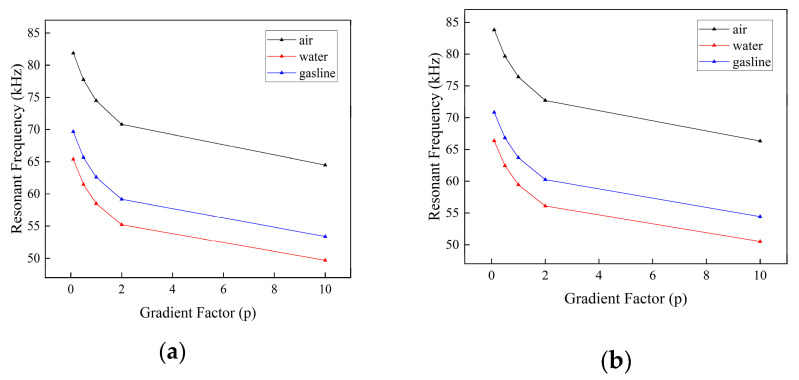
First-order resonant frequency of a sandwich micro-cantilever beam in different fluids with: (**a**) even-type porosity; and (**b**) uneven-type porosity.

From Figure 7, we can see that both the FGM sandwich micro-cantilever beam gradient factor p and the fluid environment are significantly negatively correlated for the resonance frequency.

## 4. Conclusions

In this study, utilizing the Euler-Bernoulli beam theory and the MCST, we formulated a one-dimensional heat conduction equation for functionally graded sandwich beams under photothermal stimulus. By incorporating a hydrodynamic function, we derived the analytical solutions for the thermal vibration model and the dynamic deformation field of the sandwich micro-cantilever beam in fluids. Numerical simulations were conducted to explore the impact of pore distribution, geometrical configuration, material gradient parameter, and the surrounding fluid on the vibration characteristics of the FG beams. From these analyses, we can conclude, as follows, that:

(1) The existence of fluid causes the resonance peak of the beam to slow down and the resonance frequency to migrate to lower frequencies. The higher the fluid kinematic viscosity, the more pronounced this phenomenon is;
(2) The impact of pores on the dynamic response to laser loading of the FGM cantilever sandwich beam is low compared to that of the gradient factor p and the fluid.

## Figures and Tables

**Table 1:** The material properties of the FGM sandwich cantilever beam.

Material	*E*/GPa	ν	$\rho /(kg/m^{3})$	l/μm	*K*/(W/m·K)	$\alpha_{T}/(1/K)$	*C*/(J/kg·K)
Al	70	0.3	2707	6.58	237	2.3 × 10^−7^	880
$Al_{2}O_{3}$	380	0.3	3800	11.00	30	7.4 × 10^−6^	770

Physical quantities used in this table: *E*—Young’s modulus, ν—Poisson’s ratio, ρ—density, l—scale parameter, *K*—thermal conductivity, $\alpha_{T}$—coefficient of thermal expansion, *C*—specific heat capacity.

**Table 2:** Fluid density and dynamic viscosity.

Fluids	$\rho /(kg/m^{3})$	η/(Pa·s)
air	1.205	1.81 × 10^−5^
gasoline	678	2.9 × 10^−4^
water	998	1.01 × 10^−3^

## Data Availability

The data presented in this study are available on request from the corresponding authors.
